# Diabetes Induces a Transcriptional Signature in Bone Marrow–Derived CD34^+^ Hematopoietic Stem Cells Predictive of Their Progeny Dysfunction

**DOI:** 10.3390/ijms22031423

**Published:** 2021-01-31

**Authors:** Yuri D’Alessandra, Mattia Chiesa, Vera Vigorelli, Veronica Ricci, Erica Rurali, Angela Raucci, Gualtiero Ivanoe Colombo, Giulio Pompilio, Maria Cristina Vinci

**Affiliations:** 1 Unit of Immunology and Functional Genomics, Centro Cardiologico Monzino IRCCS, 20138 Milan, Italy; yuri.dalessandra@ccfm.it (Y.D.); mattia.chiesa@cardiologicomonzino.it (M.C.); veronica.ricci@ccfm.it (V.R.); gualtiero.colombo@ccfm.it (G.I.C.); 2 Unit of Vascular Biology and Regenerative Medicine, Centro Cardiologico Monzino IRCCS, 20138 Milan, Italy; vera.vigorelli@ccfm.it (V.V.); erica.rurali@ccfm.it (E.R.); 3 Dipartimento di Medicina Clinica e Chirurgia, Università deli Studi di Napoli Federico II, 80100 Napoli, Italy; 4 Unit of Experimental Cardio-Oncology and Cardiovascular Aging, Centro Cardiologico Monzino IRCCS, 20138 Milan, Italy; angela.raucci@ccfm.it; 5 Dipartimento di Scienze Biomediche, Chirurgiche e Odontoiatriche, Università degli Studi di Milano, 20100 Milan, Italy

**Keywords:** diabetes, bone marrow, hematopoietic stem cells, CD34^+^, transcriptional profile, inflammation

## Abstract

Hematopoietic stem/progenitor cells (HSPCs) participate in cardiovascular (CV) homeostasis and generate different types of blood cells including lymphoid and myeloid cells. Diabetes mellitus (DM) is characterized by chronic increase of pro-inflammatory mediators, which play an important role in the development of CV disease, and increased susceptibility to infections. Here, we aimed to evaluate the impact of DM on the transcriptional profile of HSPCs derived from bone marrow (BM). Total RNA of BM-derived CD34^+^ stem cells purified from sternal biopsies of patients undergoing coronary bypass surgery with or without DM (CAD and CAD-DM patients) was sequenced. The results evidenced 10566 expressed genes whose 79% were protein-coding genes, and 21% non-coding RNA. We identified 139 differentially expressed genes (*p*-value < 0.05 and |log2 FC| > 0.5) between the two comparing groups of CAD and CAD-DM patients. Gene Set Enrichment Analysis (GSEA), based on Gene Ontology biological processes (GO-BP) terms, led to the identification of fourteen overrepresented biological categories in CAD-DM samples. Most of the biological processes were related to lymphocyte activation, chemotaxis, peptidase activity, and innate immune response. Specifically, HSPCs from CAD-DM patients displayed reduced expression of genes coding for proteins regulating antibacterial and antivirus host defense as well as macrophage differentiation and lymphocyte emigration, proliferation, and differentiation. However, within the same biological processes, a consistent number of inflammatory genes coding for chemokines and cytokines were up-regulated. Our findings suggest that DM induces transcriptional alterations in HSPCs, which are potentially responsible of progeny dysfunction.

## 1. Introduction

Diabetes mellitus (DM) encompasses a group of metabolic disorders characterized by hyperglycemia resulting from defects in insulin secretion, insulin action, or both. The chronic hyperglycemia of DM is associated with long-term micro- and macrovascular complications, finally resulting in end organ dysfunction and failure [1,2].

Numerous evidence indicates that both chronic inflammation and activation of the innate immune system are involved in type 1 and type 2 diabetes (T1DM and T2DM) pathogenesis, as well as in the development of related complications [3,4]. Indeed, it has been extensively documented that DM patients exhibit a chronic mild inflammatory state characterized by increased levels of circulating pro-inflammatory cytokines [5] and non-specific immunity activation, such as macrophages [6] and other innate immune cells [7,8,9,10]. Taken together, these immunological alterations are believed to contribute to the development of atherosclerosis and to the pathogenesis of DM complications, including nephropathy and retinopathy [11,12]. Consistently, anti-inflammatory therapeutic approaches have also been developed for diabetic complications [13].

Interestingly, in addition to underlying chronic inflammation, DM patients are highly susceptible to infectious diseases, often associated with impaired wound healing [14,15]. This evidence suggests that bactericidal and wound healing capacities of innate immune cells are compromised in DM context. Indeed, numerous studies in T2DM patients showed impaired phagocytic and chemotactic activity of monocytes and neutrophils [16,17,18,19], as well as decreased natural killer and dendritic cell functions [20,21].

However, the molecular mechanism underlying the paradox of innate immune system function in DM is not fully understood. Recent data suggest that hematopoietic stem/progenitor cell (HSPC) differentiation and function might be governed by metabolic reprograming at bone marrow (BM) level. In this view, dysregulated genes related to innate immune cell function could be carried down through progenitor cells to terminally differentiated cells [22].

Herein, through an innovative next generation sequencing technique (NGS), we show, for the first time, that DM promotes, at BM level, a primitive gene expression signature in HSPCs that once conveyed to differentiated cells may eventually result in the intrinsic pro-inflammatory and dysfunctional phenotype of their immune cell progenies.

## 2. Results

### 2.1. Experimental Design

We hypothesized that DM might induce persistent transcriptional alternations in HSPCs at BM level, potentially responsible for innate immune cell dysfunction of the patients. To verify this hypothesis, we performed genome wide transcriptional comparison of CD34^+^ HSPCs isolated from the sternal BM biopsies of both normoglycemic and T2DM coronary artery disease patients (CAD and CAD-DM, respectively) undergoing coronary artery bypass graft (CABG) A total of 23 patients, 11 CAD (controls) and 12 CAD-DM (cases) matched for gender and with similar age, were enrolled (Table 1).

Within this cohort, on the basis of mRNA availability, BM-CD34^+^ stem cell samples from 6 CAD and 8 CAD-DM patients were selected for genome-wide expression analysis (Table 2).

**Figure 1:**
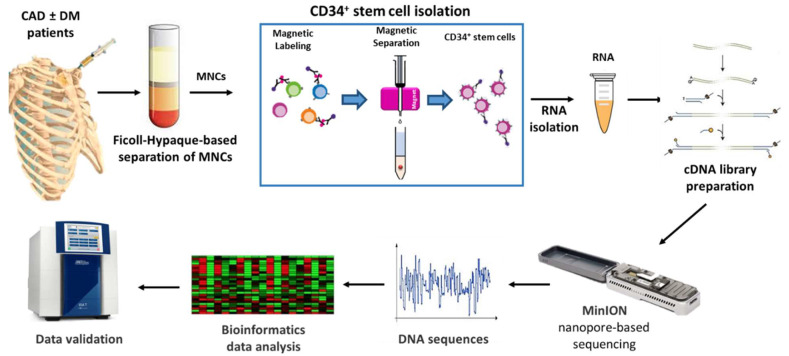
Schematic representation of the study. CAD: Coronary artery disease, DM: Diabetes mellitus, MNCs: Mononuclear cells.

Nanopore-based sequencing, as outlined in Figure 1, was used to this aim. The entire cohort of 23 patients was subsequently included in the validation phase.

### 2.2. Gene Expression Biotype in HSPCs of CAD and CAD-DM Patients

**Figure 2:**
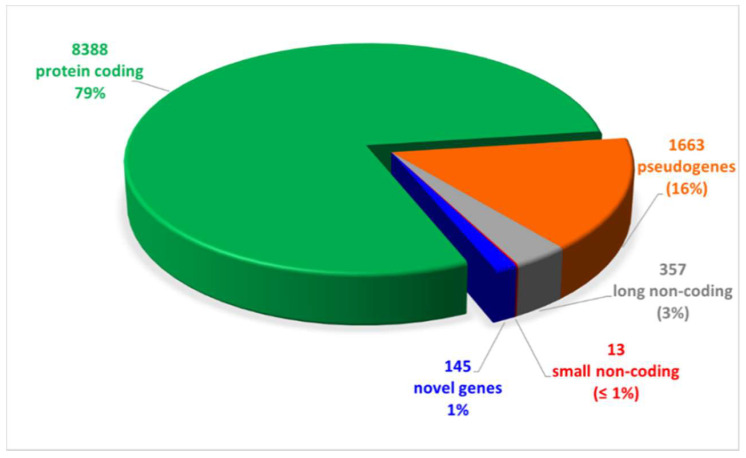
Pie chart of expressed genes, grouped by biotypes provided by the ensemble annotation. Out of 10,566 expressed genes, 79% (8388) was represented by protein coding genes. The remaining 21% was constituted by non-coding RNAs (16% pseudogenes, 3% and 1% long and small non-coding, respectively, and 1% novel genes).

We performed long-reads cDNA sequencing of samples obtained from 8 CAD-DM and 6 CAD matched patients (Table 2). The results evidenced 10,566 expressed genes, of which 79% were mRNA, while the remaining 21% were non-coding RNAs as shown in Figure 2. Notably, out of the 21% non-coding RNAs, the 76% were pseudogenes (16%) and ~ 5% novel RNA isoforms or putative novel genes (1%), namely, loci expressed in intergenic or intronic regions not yet annotated. The complete list of expressed genes, including gene symbols, locus, and relative gene expression is gathered in Table S1.

### 2.3. DM Induces a Distinct Transcriptome Profile in BM-Derived HSPCs

**Figure 3:**
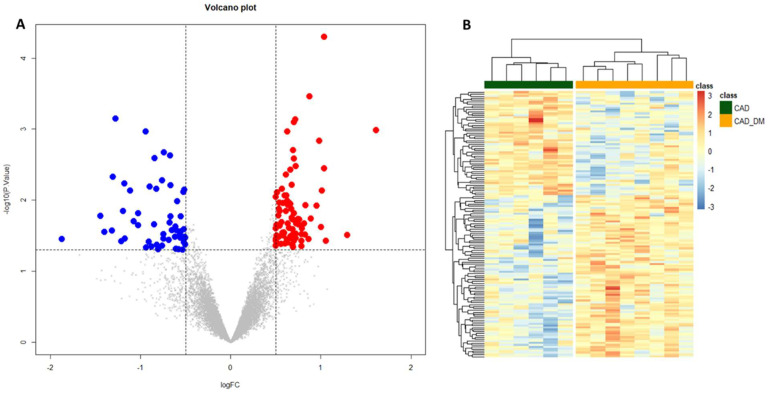
Volcano plot and Heatmap representation of differentially expressed genes. (**A**) The statistical analysis results are represented as a volcano plot of log2 fold-change (CAD-DM vs. CAD, x-axis) versus −log10 P values (y-axis). Among the 139 differentially expressed genes (*p* < 0.05), 84 genes were over-expressed (*p*-value < 0.05 and |log2 FC| > 0.5) in CAD-DM (red dots), whereas 55 in CAD (blue dots). (**B**) The heatmap shows that clustering based on the differentially expressed genes allowed complete separation between CAD-DM (orange squares) and CAD (green squares) samples. Gene expression levels were standardized and displayed as gradient colors from higher (dark orange) to lower (dark blue).

To gain insights into global gene expression modifications induced by the diabetic BM microenvironment, a differential expression analysis was performed. We identified 139 differentially expressed genes (*p*-value < 0.05 and |log2 FC| > 0.5) between the two groups of CAD and CAD-DM patients. Of these, 55 were down-regulated (blue dots), and 84 up-regulated (red dots) in CAD-DM vs. CAD, as shown in the Volcano plot of Figure 3A. Moreover, unsupervised hierarchical clustering based on differentially expressed genes revealed distinctive expression patterns within each cell type, ensuring an utter discrimination between CAD and CAD-DM samples (Figure 3B). The complete list of differentially expressed genes, including gene symbols, locus, and relative gene expression, is gathered in Table S2.

### 2.4. Functional Genomics Analysis Reveals a Disease-Specific Genomic Signature in HSPCs of CAD-DM Patients

**Figure 4:**
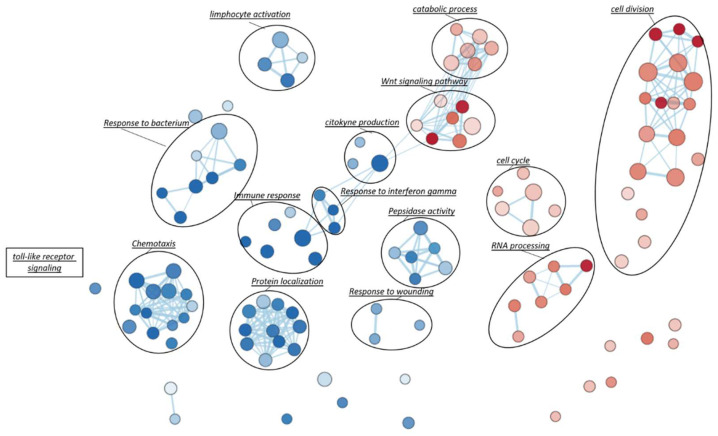
Gene-Set Enrichment Analysis (GSEA). To visually interpreting biological data from GSEA analysis, a network of the most significant Gene Ontology biological processes (GO-BP) terms (*p* < 0.05) was drawn. Blue and red nodes represent pathways mainly associated with CAD and CAD-DM conditions, respectively. The node gradient color is proportional to node significance, from lower (light node; *p* = 0.05) to higher (dark node; *p* << 0.0001); node size is proportional to the gene-set size. Edge thickness is proportional to the similarity between two gene-sets.

Gene Set Enrichment Analysis (GSEA), based on Gene Ontology biological processes (GO-BP) terms, was implemented to infer the functional roles of overrepresented genes in CAD-DM samples, allowing for the identification of disease-specific genomic signatures. Gene Ontology annotation led to the identification of fourteen overrepresented biological categories in CAD-DM samples. Remarkably, eight out of fourteen biological processes were related to lymphocyte activation, chemotaxis, peptidase activity, and innate immune response (e.g., ‘response to bacterium’, ‘toll-like receptor signaling’, ‘response to interferon gamma’, cytokine production) as shown in Figure 4. Notably, all these categories included a large proportion of downregulated genes involved in migration (i.e., CXCR4, CKLF, ITGAX, PPBP), antivirus, antifungal, and antibacterial host defense (i.e., TRIM22, TRIM5, AZU1, MPO, RNASE3, FPR1, FPR2, PPBP, DEFAs) as well as macrophage differentiation, lymphocyte emigration, proliferation, and differentiation (i.e., CSF1R, TNFSF13B, IGLL1, ADAM28, HLA-DQA1, HLA-DQB2). Importantly, within the same biological processes, a consistent number of inflammatory genes such as CCL2, IL1β, IL18RAP, CCL1, IL7, CCL3L1, CCL13, CCL4, NFKB1, and ADAM17 resulted in being upregulated. Interestingly, besides immune system categories, biological processes related to cell division, cell cycle, catabolic process, RNA processing and WNT signaling pathways also resulted as overrepresented in BM-CD34^+^ HSPCs of CAD-DM patients, suggesting a direct impact of DM on HSPC proliferation, metabolism, and differentiation. The lists of the genes representative of each biological category displayed in the Figure are gathered in Table S3. Moreover, all the processes identified by GSEA related to the downregulated and the upregulated genes are reported in Table S4 and Table S5, respectively.

### 2.5. Validation of Sequencing Data with qPCR

**Figure 5:**
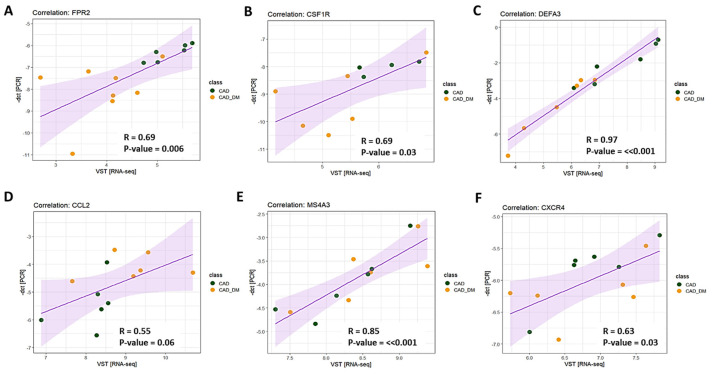
Technical validation of gene expression. The expression level of 6 genes was assessed using RT-qPCR single assays and RNA-seq. Pearson’s correlation coefficient (R) was computed to evaluate the strength of association between the two methodologies. The 95% confidence interval of the trendline (purple line) is depicted in light purple. Data are plotted as -dct (y-axis) versus log-normalized value (x-axis) for FPR2 (panel **A**), CSFR1 (panel **B**), DEFA3 (panel **C**), CCL2 (panel **D**), MS4A3 (panel **E**), and CXCR4 (panel **F**). The R coefficient as well as the corresponding *p*-values for each gene are shown in the relative panel.

To validate results obtained with sequencing analyses, we selected 6 genes involved in chemotaxis, immune response, and host defense to further assess their expression levels by qPCR. According to RNA sequencing, we chose three top differentially expressed genes (i.e., FPR2, CSFR1, and DEFA3), and three that showed the tendency to be up- and down modulated (i.e., CCL2, MS4A3, and CXCR4) to strength the appropriateness of our global transcription analysis. Then, we compared the expression levels obtained by qPCR and RNA-Seq for each selected gene. The analysis displayed a strong and significant correlation for all tested genes (Figure 5).

**Figure 6:**
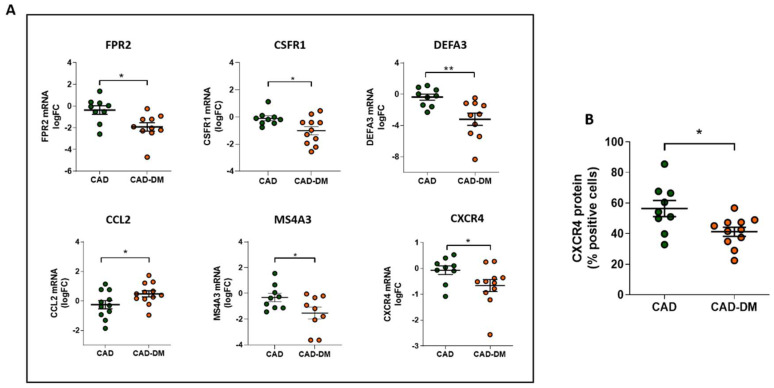
**Panel A:** Analysis of FPR2, CSFR1, DEFA3, CCL2, MS4A3, and CXCR4 mRNA expression in CD34^+^ HSPCs from CAD and CAD-DM by qPCR. Data are expressed as log2 fold-change (logFC). CAD has been used as controls (* *p* < 0.05; ** *p* < 0.01 vs. CAD; unpaired *t* test). **Panel B**: CXCR4 membrane expression level in CD34^+^ HSPCs from CAD and CAD-DM by flow cytometry (* *p* < 0.05 vs. CAD; unpaired *t* test). Data are expressed as percentage of positive cells.

Notably, we further corroborated our results on mRNA of BM-CD34^+^ stem cell samples isolated from patients who were not included in the sequencing cohort but originally enrolled (Table 1). In line with sequencing data, qPCR analysis confirmed the same expression trend that was statistically significant for all selected genes (Figure 6, panel A). Importantly, the analysis by flow-cytometry of CXCR4 protein expression, a gene that by sequencing displayed a down-regulation trend, also demonstrated a significant reduction of the receptor at membrane level (Figure 6, panel B).

## 3. Discussion

There is a close link between DM and atherosclerotic CV disease, which remains the most prevalent cause of morbidity and mortality in diabetic patients. The exact pathogenic mechanisms linking accelerated atherosclerotic cardiovascular complications, resulting in long-term damage and failure of various organ systems, and diabetes, are rather complex [23]. However, it is widely reported that in all stages of atherosclerosis, both in the absence and presence of diabetes, monocytes and macrophages are key players [24]. Preclinical and clinical studies support the role of inflammation in the initiation and progression of atherosclerosis that despite representing the intrinsic capacity of the body to react to tissue injury or pathogens can have detrimental effects on tissue and organs, if chronically activated [25,26]. To this regard, a chronic low-grade inflammation and immune activation have been described both in pre-diabetic and diabetic states [27,28,29,30]. A consisting body of literature suggests that the impact of hyperglycemia on HSPCs in the BM niche may be the primary factor contributing to CV disease development [31,32]. Indeed, numerous preclinical data show the ability of BM diabetic environment to boost HSPC differentiation toward myeloid lineage as well as to promote abnormal generation and accumulation of immune cell subpopulations (e.g., monocytes and macrophages) with more aggressive phenotypes [33,34,35]. Paradoxically, this state of chronic inflammation, characterized by increased myelopoiesis and circulating inflammatory cytokines, which account for increased risk of atherosclerosis, coexists with a dysfunctional immune response that renders diabetic patients more susceptible to infections [36].

Interestingly, though the molecular mechanisms underpinning the paradox of immune system function in DM is not fully understood, preclinical data suggest that epigenetic modifications in HSPCs triggered by diabetic BM microenvironment can, once passed to their progeny, be responsible for the gene expression and phenotypical alteration of terminally differentiated immune cells [22]. Herein, in order to explore if a transcriptional signature predictive of immune cell dysfunction was already present in HSPCs of DM patients, we compared the transcripts of CD34^+^ HSPCs isolated from sternal BM biopsies of CAD patients with or without T2DM by genome-wide expression analyses. The study involved the use of nanopore-based sequencing, a new NGS technique that allowed us to obtain a complete picture of RNA transcripts, specifically 79% mRNA and 21% non-coding RNA. Succeeding analysis of the data by the Gene GO-BP-based GSEA, revealed fourteen overrepresented biological categories in CAD-DM vs. CAD samples. Remarkably, most of the relevant biological processes were related to immune system function, namely ‘chemotaxis’, ‘response to bacterium’, ‘toll-like receptor signaling’, ‘response to interferon gamma’, cytokine production, and response to bacterium. In addition, narrowing our analysis down within the gene lists of biological processes, we found a down-regulation of numerous genes coding for cytotoxic peptides related to innate immune system with bactericidal, antifungal, and antiviral properties. These included AZU1, a neutrophil- monocyte-derived antibacterial and chemotactic glycoprotein with cytotoxic action against Gram-negative bacteria [37], defensins family members (i.e., DEFA1, DEFA1B, DEFA3, and DEFA4), abundant in the granules of neutrophils, as well as MPO and RNASE3, additional genes encoding for proteins with microbicidal activity against a wide range of organisms [38,39,40]. Notably, CD34^+^ HSPCs from CAD-DM patients also displayed a defective expression of genes involved in the regulation of macrophage differentiation (CSFR1), phagocytic cell recruitment and activation (i.e., PPBP, CXCR4, and FPR2), as well as antigen presentation (i.e., HLA-DQA1 and HLA-DQB2) [41]. Consistently in DM, despite increased myelopoiesis, there is a defense mechanism impairment of granulocytes and monocytes including migration to the site of inflammation, phagocytosis, ROS production, and germicidal activity. The functional failure of these cells, which are the first cells recruited to the sites of injury, plays a crucial role in inflammatory response against microbial infections and it is associated with an increased incidence of infections in DM patients [42].

However, such a gene expression pattern in CD34^+^ HSPCs, predictive of innate immune system impairment, was associated with upregulation of numerous genes encoding for pro-inflammatory interleukins, chemokine, and receptors, critical not only in the recruitment, accumulation, and activation of immune cells to the site of injury, but also in all inflammatory stages of atherosclerosis [42,43]. Among these, it is noteworthy mentioning CCL13, a chemotactic factor able to identify with a very high degree of accuracy subjects with clinically significant atherosclerotic heart disease [44]. The upregulation of pro-inflammatory genes in HSPCs of CAD-DM patients strongly suggests the acquisition of senescent-associated secretory phenotype (SASP). Similar to aging, diabetes is known to induce cell senescence with mechanisms involving, together with others, mitochondrial dysfunction and increased reactive oxygen species [45]. These do not spare immune cells and stem/progenitor cells that, as all other cells, are susceptible to developing premature senescence [45,46]. SASP profile, characterized by sustained release of pro-inflammatory cytokines, chemokines, and growth factors, may in turn, with a vicious positive feedback cycle, contribute to establishing a chronic low-grade pro-inflammatory status [47], and exacerbate immune dysfunction [45,48].

Despite the limitation of the study related to the particularity of biological samples that constrains the number of subjects, to our knowledge this is the first study revealing the gene expression profile of BM-derived CD34^+^ HSPCs from CAD patients who are distinguished by the only presence of T2DM. In line with recent evidence demonstrating the ability of DM to induce stable epigenetic modifications responsible for gene expression alteration in BM progenitors [22,49], our study suggests that diabetic BM microenvironment, regardless of the combination of comorbid CAD, establishes a transcriptional signature in HSPCs that is predictive of unrestrained inflammatory and dysfunctional phenotype of their daughter cells in the peripheral tissues. Epigenetic aberrant modifications, which are cell-type-specific and can be mitotically heritable contributing to long-term gene expression changes, are associated with many human diseases, including diabetes. Numerous studies already provide evidence that DNA methylation and post-translational modifications of histone proteins are involved in the development of diabetes and its vascular complications [50]. Differences in activator (e.g., H3K9ac, H3K4me) and repressive (e.g., H3K36me3, H3K9me3) chromatin marks have been found at the level of pro-fibrotic and inflammatory genes in numerous tissues [51,52], including immune cell components (i.e., monocytes and lymphocytes) of diabetic patients [53,54]. While it is becoming evident that diabetes, by glucose metabolism alteration and oxidative stress (ROS), can affect the function of epigenetic machinery [55,56], big gaps still exist in knowledge about the interplay between cell signaling and epigenetic machinery. However, as epigenetic enzymes can regulate cell signaling pathways (e.g., NF-κB, RAS/RAF/MEK/MAPK, PI3K/Akt, Wnt/β-catenin, p53, and ERα) [57], it is very likely that signal transduction pathways and transcription factors abnormally activated by diabetic conditions or hyperglycemia can cooperate, in a still not fully elucidated way, with epigenetic factors to promote sustained expression of pathological genes [58]. To this regard, we are currently studying the molecular and epigenetic mechanisms involved in glucose-induced dysfunction of HSPCs.

Overall, our results may open the way to further studies aimed at unravelling the profound mechanisms underlying the paradox of innate immune training in diabetes with the finally scope of discovering novel therapeutic targets to be exploitable for limiting and/or preventing infectious diseases and devastating complications in DM patients.

## 4. Materials and Methods

### 4.1. Study Participants

Twenty-three patients, 11 CAD and 12 CAD-DM, were enrolled in the study. All procedures performed on subjects were in accordance with the Helsinki Declaration of 1975. The investigation was approved by Centro Cardiologico Monzino Research Ethics Committee (No. CCM 205–RE1973/1) and each participant provided written informed consent. The inclusion criteria for CAD-DM patients were age above 35 years, diagnosis of T2DM as defined by the American Diabetes Association [59], with at least one year of disease duration at the time of the screening visit. The exclusion criteria were T1D diagnosis, inflammatory/infective/autoimmune disorders, and/or history of cancer. At admission, CAD-DM patients were treated with any combination of oral anti-diabetic therapies with/without insulin.

### 4.2. Sternal Bone Marrow Biopsy and CD34^+^ Stem Cell Isolation

During surgical procedure, two or three milliliters of sternal BM were withdrawn by bone biopsy needle (15G × 25/90mm; MDL). Blood aspirate was suspended in saline buffer, and mononuclear cell (MNC) fraction was isolated by density gradient centrifugation using Lymphoprep^TM^ (Sentinel Diagnostic Spa; Milan, Italy). CD34^+^ stem cells were then magnetically sorted by MiniMACS system (CD34 Microbead Kit; Miltenyi Biotec GmbH; Bologna, Italy). Purity of isolated cells was assessed during experimental settings by flow cytometry (Beckman-Coulter Gallios, Life Science; Milan, Italy). The test was carried out three times on samples deriving, randomly, from both cohort of patients. Isolated cells were single stained for the hematopoietic stem cell markers CD34 and for CD14, CD3, CD80, and CD86 to determine the level of cell lineage contamination. The 90% ± 4.08 (SD) of the cells resulted positive for CD34 and negative for all other considered markers (Figure S1). Isolated CD34^+^ cells were then cryopreserved for subsequent RNA isolation and analysis.

### 4.3. Total RNA Extraction

Total RNA from CAD and CAD-DM CD34^+^ cells was isolated using the Direct-zol RNA Kit (Zymo Research; EuroClone S.p.A., Milan, Italy) following the manufacturer’s instructions. At the end of the procedure RNA concentration and quality were assessed, respectively, by microvolume spectrophotometry using a ND-1000 NanoDrop (Thermo Fisher Scientific; Milan, Italy) and by microfluidics-based automated electrophoresis, using the RNA 6000 Nano Assay Kit on 2100 BioAnalyzer system (Agilent Technologies S.p.A.; Milan, Italy).

### 4.4. MinION Nanopore Sequencing

RNA sequencing was performed on 6 CAD and 8 CAD-DM samples selected on the basis of RNA amount availability using a MinION Mk1B sequencer (Oxford Nanopore Technologies; Oxford, UK) on a R9.4.1 (FLO-MIN106) flow cell (Oxford Nanopore Technologies; Oxford, UK). Clinical characteristics of the sequenced patients are shown in Table 2. The libraries were prepared using the SQK-LSK108 Sequencing kit (Oxford Nanopore Technologies; Oxford, UK) following the 1D Strand switching cDNA-by-ligation protocol. Briefly, reverse transcription was performed starting from 500 ng of RNA using the PCR-VN-RT primer and the Strand Switch Oligo PCR_SW_mod_3G, followed by First Strand cDNA PCR amplification for full-length transcripts (18 cycles, 3 min extension). After purification by Agentcourt AMPure XP beads (Beckman Coulter), end-repairing and dA tailing steps were performed on 1 μg of DNA/sample before the ligation of specific adapters to 0.2 pmols of end-prepped DNA. A single library was loaded on each flowcell following protocol instructions. After reaching at least 5 GB of acquired data, the sequencing was stopped and the flowcell washed in order to allow the loading of a new sample. Then the flow cell underwent QC analysis to verify the presence of enough pores to run the sequencing. In case of insufficient pores availability, a new flow cell was loaded. Albacore software v2.3.1 (Oxford Nanopore Technologies; Oxford, UK) was used for basecalling.

### 4.5. Bioinformatics and Statistical Analysis

Reads were mapped to the human genome reference GRCh38.96 by the ‘minimap2′ aligner, properly set for minION long reads (custom options: -x map-ont -Y) [60]. Genes were considered as “expressed” when showing a minimum of 10 counts in at least 50% of samples. Gene expression quantification was performed by ‘featureCount’ (custom option: -L) [61], while gene filtering and variance-stabilizing normalization (VSN) were carried out by the ‘DaMiRseq’ R package [62]. The statistical analysis was accomplished by the ‘limma’ R package [63]. The Benjamini–Hochberg procedure was used to control for the false discovery rate (FDR). Differences were deemed significant if the *p*-value was < 0.05 and the |log2 FC| > 0.5. Clustering analysis was performed by the ‘pHeatmap’ R package. Demographic and clinical profile of enrolled patients are expressed as mean ± standard deviation, median [25–75% confidence interval], or percentage, as appropriate. Shapiro–Wilk normality test has been used to verify Gaussian distribution of the considered covariates. For comparison between two groups, Student’s T or Mann–Whitney U test have been performed, as appropriate. Fisher’s exact test has been used to compare categorical covariates. Dot-plots of qPCR validation were drawn by GraphPad Prism (v.5, GraphPad Software,) and given as mean ± SEM. All data expressed as fold-change were log2-transformed before analysis and tested for the normality by using the Shapiro–Wilk normality test. Differences between data were evaluated by unpaired Student’s t test (2-group comparisons).

### 4.6. Functional Analysis

The Gene-Set Enrichment Analysis (GSEA v.4.0.0) with pre-ranked mode was performed to infer biological function, associated with CAD and CAD-DM phenotypes [64]; the ranking metrics was based on the t-statistics values of differential expression analysis (only protein coding genes) and only the gene ontology biological process (GO-BP) were taken into account. Pathways significantly associated with phenotypes (*p* < 0.05) were visually represented by the Enrichment Map (v.3.0.0) Cytoscape (v3.7.1) plug-in [65,66].

### 4.7. qPCR Validation

The gene expression levels were technically validated by qPCR on 6 genes (i.e., FPR2, CSFR1, DEFA3, CCL2, MS4A3, and CXCR4). The validation was performed on samples obtained from the entire cohort of 23 CAD and CAD-DM patients (Table 1). Total RNA (400 ng) was reverse-transcribed using 5x-All-in-One RT Master Mix (Applied Biological Materials; Gentaur, Bergamo, Italy) and the resulting cDNA diluted to 5 ng/μL with RNASe free water. Single assay Real Time qPCR was performed using specific Taqman Gene Expression Assays (Thermo Fisher Scientific; Milan, Italy) for each target, according to manufacturer’s instructions, and run on Viia 7 RT-qPCR system. Ct values were normalized (Delta Ct), using beta globulin as reference gene. Data, expressed as fold-changes (FC; 2^−ΔΔCT^) over CAD after normalization to each housekeeping gene, were log2-trasformed before analysis. The Pearson’s correlation indexes and the corresponding *p*-values of sequenced samples were calculated by the native R base functions (v.3.6.1) [67] and represented by the ‘ggplot2′ R package [68].

### 4.8. Flow Cytometric Assay

The purity of the cells and protein expression of non-significantly downregulated gene (CXCR4) was validated by flow-cytometry. CD34^+^ stem cells were single stained for 30 min with antihuman CD34, CD14, CD3, CD80, CD86, and CXCR4 monoclonal antibodies (BD Biosciences). After 30 min, cells were washed and analyzed. The Beckman-Coulter Gallios platform (Beckman-Coulter Life Science; Milan, Italy) and Kaluza analysis software (v2.1.1) were used to analyze samples by use of appropriate physical gating. At least 10^4^ events in the indicated gates were acquired.

## Figures and Tables

**Table 1:** Clinical profile, cardiovascular (CV) risk factors and therapies characterizing the coronary artery disease (CAD) cohort enrolled for this study. Patients have been categorized for the presence of diabetes mellitus (DM) as comorbidity.

	CAD	CAD-DM	*p*-Value
*N*	11	12	
Gender (male)	91%	92%	*1.000*
Age (years)	70 [57–77]	70 [65–74]	*0.916*
BMI (Kg/mq)	28.05 ± 4.04	27.01 ± 3.87	*0.536*
Glycemia (mg/dL)	111 ± 14	155 ± 49 °	*0.015*
LDL (mg/dL)	136 [93–157] ^#^	75 [72–89] °	*0.018*
HDL (mg/dL)	44.3 ± 9.2 ^#^	47.3 ± 12.0 °	*0.536*
Total cholesterol (mg/dL)	190.2 ± 43.2 ^#^	156.1 ± 34.2 °	*0.058*
Creatinine (mg/dL)	0.92 [0.88–1.05]	1.03 [0.85–1.24]	*0.338*
**Other CV Risk Factors**			
Hypertension	82%	100%	*0.217*
Dyslipidemia	73%	67%	*1.000*
Smoke	18%	42%	*0.370*
**DM Therapies**			
Oral antidiabetic agents	0	83%	
Insulin	0	8.5%	
Oral antidiabetic agents + insulin	0	8.5%	
**Other Therapies**			
Antihypertensive drugs	100%	92% °	*1.000*
Lipid-lowering drugs	45%	75%°	*0.214*

# Data available for 10 out of 11 patients, ° data available for 11 out of 12 patients.

**Table 2:** Clinical profile, CV risk factors, and therapies characterizing the subgroup of CAD patients selected for RNA sequencing. Patients have been categorized for the presence of DM as a comorbidity.

	CAD	CAD-DM	*p*-Value
*N*	6	8	
Gender (male)	100%	100%	*1.000*
Age (years)	66.3 ± 11.6	70.1 ± 5.36	*0.427*
BMI (Kg/mq)	26.80 ± 3.26	26.31 ± 3.10	*0.782*
Glycemia (mg/dL)	120 ± 15 ^#^	162 ± 44 °	*0.049*
LDL (mg/dL)	134 [70–146] ^#^	75 [70–85] °	*0.287*
HDL (mg/dL)	43.0 ± 10.4 ^#^	48.7 ± 12.5 °	*0.424*
Total cholesterol (mg/dL)	176.8 ± 48.5 ^#^	151.6 ± 39.8 °	*0.345*
Creatinine (mg/dL)	0.94 [0.90–1.06]	0.98 [0.85–1.24]	*0.880*
**Other CV Risk Factors**			
Hypertension	83%	100%	*0.429*
Dyslipidemia	67%	63%	*1.000*
Smoke	0%	50%	*0.085*
**DM Therapies**			
Oral antidiabetic agents	0	87.5%	
Insulin	0	0	
Oral antidiabetic agents + insulin	0	12.5%	
**Other Therapies**			
Antihypertensive drugs	100%	88%	*1.000*
Lipid-lowering drugs	50%	75%	*0.580*

# Data available for 5 out of 6 patients, ° data available for 7 out of 8 patients.

## Data Availability

Data is contained within the article or Supplementary Material.

## Supplementary Materials

Supplementary materials can be found at https://www.mdpi.com/1422-0067/22/3/1423/s1.

## Abbreviations

BM	Bone marrow
CABG	Coronary artery bypass surgery
CAD	Coronary artery disease
CV	Cardiovascular
DM	Diabetes mellitus
FDR	False discovery rate
GO-BP	Gene Ontology-biological processes
GSEA	Gene set enrichment analysis
HPSCs	Hematopoietic stem/progenitor cells
MNC	Mononuclear cell
NGS	Next generation sequencing
T1DM	Type 1 diabetes mellitus
T2DM	Type 2 diabetes mellitus
VSN	Variance-stabilizing normalization
