# Supplementary figures and images for: Diabetes Induces a Transcriptional Signature in Bone Marrow–Derived CD34+ Hematopoietic Stem Cells Predictive of Their Progeny Dysfunction
Source: Int J Mol Sci. 2021 Jan 31;22(3):1423. doi: 10.3390/ijms22031423 (PMC7866997; doi:10.3390/ijms22031423)

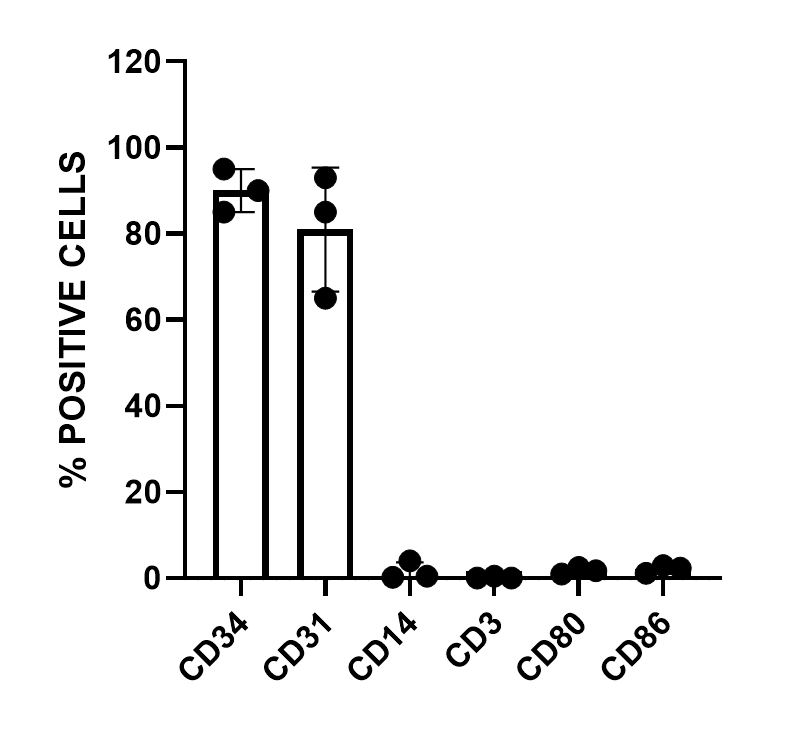

Supplement: Supplementary file 1 [file ijms-22-01423-s001.zip › SUPPLEMENTARY/Figure S1.tif]
